# Reading Emotions from Body Movement: A Generalized Impairment in Schizophrenia

**DOI:** 10.3389/fpsyg.2015.02058

**Published:** 2016-01-14

**Authors:** Anja Vaskinn, Kjetil Sundet, Tiril Østefjells, Katharina Nymo, Ingrid Melle, Torill Ueland

**Affiliations:** ^1^Department of Psychology, University of OsloOslo, Norway; ^2^Norwegian Centre for Mental Disorders Research KG Jebsen Centre for Psychosis Research, Oslo University HospitalOslo, Norway; ^3^Division of Mental Health Services, Department for Specialized Inpatient Treatment, Akershus University HospitalLørenskog, Norway; ^4^Department of Mental Health and Addiction, Institute of Clinical Medicine, University of OsloOslo, Norway

**Keywords:** social cognition, body language, schizophrenia, sex, gender, biological motion, emotion perception

## Abstract

Body language reading is a social cognitive process with importance for successful maneuvering of social situations. In this study, we investigated body language reading as assessed with human point-light displays in participants with a diagnosis of schizophrenia (*n* = 84) compared to healthy control participants (*n* = 84), aiming to answer three questions: (1) whether persons with a diagnosis of schizophrenia have poorer body language reading abilities than healthy persons; (2) whether some emotions are easier to read from body language than others, and if this is the same for individuals with schizophrenia and healthy individuals, and (3) whether there are sex differences in body language reading in participants with schizophrenia and healthy participants. A fourth research aim concerned associations of body language reading with symptoms and functioning in participants with schizophrenia. Scores on the body language reading measure was first standardized using a separate sample of healthy control participants (*n* = 101). Further results showed that persons with schizophrenia had impaired body language reading ability compared to healthy persons. A significant effect of emotion indicated that some emotions (happiness, neutral) were easier to recognize and this was so for both individuals with schizophrenia and healthy individuals. There were no sex differences for either diagnostic group. Body language reading ability was not associated with symptoms or functioning. In conclusion; schizophrenia was characterized by a global impairment in body language reading that was present for all emotions and across sex.

## Introduction

Reading social and emotional cues and drawing inferences about another person's mind, often referred to as social cognition, is a constant human activity of considerable importance. Our social cognition helps us understand other people and when done successfully, enables us to predict what others might do next. It involves decoding people's emotional expressions (from the face, the voice, or the body), and understanding the reasons and rules for social interactions.

Humans need very little information in order to infer others' emotions and personality traits. For example, social meaning is often attributed when there is hardly any present. This phenomenon is illustrated in tasks using geometrical figures moving around inside a box such as the Animated Triangles Task (Abell et al., 2000). Even though the stimuli consist of simple geometrical shapes, their movement is fastly inferred in terms of human (inter)action. For example, one figure moving after the other is easily interpreted as one chasing the other. The human tendency to pick up social and emotional cues from limited information is also evident in point-light display (PLD) tasks. In PLD tasks (Johansson, 1973) a light is connected to different parts of the human body, or the face, while the person is filmed when moving in a dark room. In this methodology, all information except that of biological motion is eliminated, enabling investigation of pure motion, devoid of shape or color. PLDs are one way to operationalize body language reading. Perceivers are able to infer sex (Kozlowski and Cutting, 1977), personality traits (Gunns et al., 2001), and emotions from PLDs, and a few studies have shown better visual sensitivity and detection of angry PLD walkers than PLD walkers displaying other emotions (Chouchourelou et al., 2006; Ikeda and Watanabe, 2009). One possible explanation for this is that upon detecting anger, we need to act to get away from a potentially dangerous person (Ikeda and Watanabe, 2009). A neuroanatomical explanation of this phenomenon focuses on the interconnections between the superior temporal sulcus (STS)—which is responsive to human movement (Beauchamp et al., 2002), including the movement in PLDs (Grossmann et al., 2000)—and the amygdala, which keeps track of threatening stimuli and danger (Amaral et al., 2003). Angry stimuli will engage the amygdala, which again further stimulates the STS; this link is thought to support the enhanced detection of angry PLD walkers (Chouchourelou et al., 2006).

Social cognition is reduced in a number of developmental and neuropsychiatric disorders, and it has been suggested that deficits in biological motion perception may underlie the social deficits seen in these conditions (Pavlova, 2012). One of these disorders is schizophrenia, where social cognition is among the strongest predictors of functioning (Fett et al., 2011). Social cognition in the schizophrenia field is defined as emotion processing, theory of mind, social perception, and attributional style (Pinkham, 2014) with large deficits present for all domains compared to healthy control participants (Savla et al., 2013). Social cognitive processes in schizophrenia have been divided into a bottom-up perception of emotions type of process on the one hand, and a higher level top-down process of inferring mental states on the other. Body language reading from PLDs is a bottom-up process that can be placed under emotion processing. PLD tasks have been used in a few previous schizophrenia studies. In a study that used facial PLDs, schizophrenia participants were better at recognizing emotions from moving as opposed to static images, but still performed significantly worse than healthy control participants (Tomlinson et al., 2006). Another study examined the ability of individuals with schizophrenia to decide whether two whole-body PLDs were interacting or acting independently of each other (Okruszek et al., 2015). The schizophrenia group was impaired, mostly due to misclassification of independent situations as interactions. Couture et al. (2010) used stimuli of human PLD walkers developed by Heberlein et al. (2004) and found schizophrenia participants to be impaired compared to healthy control participants. These PLD walker stimuli represent body language reading stimuli of an actor moving in a dark room displaying one of four emotions (anger, happiness, sadness, fear) or neutral. The test has been referred to as Emotional Biological Motion (EmoBio). Henry et al. (2010), using the same stimuli, found participants with schizophrenia to be impaired only in recognition of fear relative to healthy control participants. Schizophrenia participants are also impaired in extracting social information from bodily emotional cues of digital walkers (Peterman et al., 2014) and in the recognition of emotions from static (Van den Stock et al., 2011) and dynamic whole-body expressions (Strauss et al., 2015) where no facial information is present. Most studies have found a general deficit in body language reading for all emotions. However, one study found a specific deficit for fear (Henry et al., 2010), another for happiness and sadness (Strauss et al., 2015). Further studies of the effect of specific emotions on body language reading are needed to provide more conclusive evidence.

There is data to indicate that women with schizophrenia perform better than men with schizophrenia on emotion perception tasks not involving bodily stimuli (Scholten et al., 2005; Van't Wout et al., 2007; Vaskinn et al., 2007) corroborating findings from healthy samples where the same sex effect is seen (Thayer and Johnsen, 2000; Hall and Matsumoto, 2004). Less is known about sex differences in the reading of emotions from body language in schizophrenia. Of the studies reviewed above only Strauss et al. (2015) examined the effect of sex on body language reading. They found a “different sex difference” among participants with schizophrenia. In the schizophrenia group, women were less accurate than men, whereas the opposite pattern was seen for healthy control participants. None of the studies using PLD stimuli examined sex effects. For healthy individuals however, sex differences have been documented for PLD tasks (Alaerts et al., 2011) with females outperforming males. However, more detailed studies indicate that the sex effect might depend upon the emotion displayed. Sokolov et al. (2011) used PLDs of door-knocking with angry, happy or neutral motion and found an interaction effect of sex by emotion. Female observers were better at recognizing neutral knocking and tended to have better recognition of angry knocking, whereas male observers excelled at recognizing happy knocking. In a follow-up study by the same group (Krüger et al., 2013) the sex of the actor was included in the equation. Again, male observers were better than female observers at recognizing happy knocking, but only when the actor was female. Female observers had a tendency to outperform male observers with regard to recognizing angry knocking from male actors. It is unknown if similar sex effects are present for body language reading from PLDs in persons with schizophrenia.

The EmoBio test was included as one of several paradigms taken from social neuroscience in the recent National Institute of Mental Health-sponsored study “Social Cognition and Functioning in Schizophrenia” (SCAF). The study sought to establish if measures from social neuroscience could constitute reliable and valid endpoints in clinical trials in schizophrenia (Green et al., 2013). The SCAF study found EmoBio to discriminate well between participants with schizophrenia and healthy participants, and it was well-tolerated (Kern et al., 2013), but, like the other measures, showed limited correlations with measures of functioning and symptoms, indicating somewhat restricted external validity (Olbert et al., 2013). Whereas, the SCAF study suggests that EmoBio might not be the best choice for a clinical trial, it may still provide knowledge about the nature of social cognitive impairments in schizophrenia. It may shed light on emotion-specific impairments, as well as the presence of sex differences in social cognitive abilities in schizophrenia.

In summary, the question of whether the emotion perception deficit in body language reading in schizophrenia is specific or general, and to what extent it depends on sex, remains unresolved. Further, a limitation of previous studies is small sample sizes (ranging from 16 to 44 participants with schizophrenia). The present study set out to resolve some of these issues and is the first to investigate the effect of sex as well as of specific emotions on body language reading from PLDs in a large sample of healthy participants and participants with schizophrenia. We have three research aims. First, we ask whether participants with schizophrenia are impaired in body language reading. Second, we ask if some emotions are easier to recognize, and if so, whether this is the same for participants with schizophrenia as for healthy control participants. Third, we investigate if there are sex differences in body language reading, and if so, whether they are present in participants with schizophrenia as well as in healthy control participants. Finally, symptomatic and functional correlates of body language reading in participants with schizophrenia are investigated. We expect associations with symptoms to be small and non-significant, but associations with functioning to be stronger.

## Materials and methods

### Participants

The study was conducted within the multi-center Thematically Organized Psychosis (TOP) Study at NORMENT KG Jebsen Centre for Psychosis Research at the University of Oslo in Norway from 2010 to 2014. Participants with schizophrenia (*n* = 60) or schizoaffective disorder (*n* = 24) according to the Diagnostic and Statistical Manual for Mental Disorders (American Psychiatric Association, 1994) were recruited from hospitals in the Oslo area. Healthy control participants (*n* = 185) from the same geographical areas were recruited through national statistical records, invited by letter to participate and screened with an interview to capture symptoms of severe mental illness (Primary Care Evaluation of Mental disorders; PRIME-MD; Spitzer et al., 1994). Healthy control participants were excluded from the study if mental, neurological or somatic disorder was confirmed or suspected. Of the 185 healthy control participants, the 101 first consecutively recruited (61 males/40 females) were used as a reference sample in order to standardize the EmoBio test in a Norwegian context. The next 84 healthy control participants that were recruited were used in the analyses of the study's research aims. These two healthy control groups did not differ in age (*t* = −0.845, *p* = 0.399) or sex distribution (*x*^2^ = 0.002, *p* = 0.965).

All participants had an IQ ≥ 70 as assessed with the Wechsler Abbreviated Scale of Intelligence (WASI; Wechsler, 2007). Participants with schizophrenia were tested in a clinically stable state to ensure that symptomatology did not interfere with test performance. Clinical features were assessed with the Positive and Negative Syndrome Scale (PANSS) (Kay et al., 1987) and the split Global Assessment of Functioning for symptoms (GAF-s), and functioning (GAF-f) (Pedersen et al., 2007). The scores on these instruments confirm a clinically stable state at time of assessment. See Table 1 for demographic and clinical information.

**Table 1:** **Demographics in participants with schizophrenia (SZ) and healthy participants (HC), and clinical features in participants with schizophrenia (SZ)**.

	**SZ (*n* = 84)**	**HC (*n* = 84)**	**Statistic**	
	**Mean (*SD*)**	**Mean (*SD*)**	**Value**	**Sig**
Age	29.0 (8.7)	30.8 (8.1)	*t* = 1.35	ns
Gender (males/females)	53/31	51/33	x^2^ = 0.10	ns
WASI IQ	99.8 (13.6)	112.0 (11.5)	*t* = 6.32	*p* < 0.001
GAF-symptoms	43.3 (11.6)^*^	–	–	–
GAF-function	44.1 (11.7)^*^	–	–	–
PANSS positive	14.5 (4.6)^*^	–	–	–
PANSS negative	14.6 (5.1)^*^	–	–	–

*WASI, Wechsler Abbreviated Scale of Intelligence; GAF, Global Assessment of Functioning; PANSS, Positive and Negative Syndrome Scale*.

* *n = 80 due to missing data*.

The study is approved by Norway's Regional Ethics Committee South East (REC South East), and is completed in accordance with the Helsinki Declaration. All participants received oral and written information on the study and have signed informed consent.

### Stimuli

We used Heberlein et al's (2004) stimuli, as adapted by Couture et al. (2010). This EmoBio version is comprised of 22 short clips of PLD walkers. The clips were shown on a computer screen, and participants indicated on a piece of paper which emotion was depicted by ticking the right box. Emotions were angry, happy, sad, fearful, or neutral/no emotion. We used the proportional scoring method used by previous studies (Heberlein et al's, 2004; Couture et al., 2010) where each response is given credit based on the proportion of healthy control participants giving that response. If 60% of healthy control participants say “happy,” 25% say “sad” and 15% say “neutral,” a “happy” response is scored 1 (60/60), a “sad” response is scored 0.42 (25/60), and a “neutral” response is scored 0.25 (15/60). With such a scoring method a certain degree of variability is accepted as normal. We based the scoring on the distribution of scores in our healthy reference sample (*n* = 101), i.e., using Norwegian norms. The distribution of responses in this standardization sample is reported in Table 2. For the majority of the PLD movie clips agreement in the healthy reference sample is quite substantial. Both the overall total EmoBio score as well as the EmoBio scores for each of the five emotion categories are used in the current study. Results can be seen in Table 3.

**Table 2:** **Distribution of responses (percentage) on the 22 point-light walker movie clips in the healthy standardization sample (*n* = 101) with resulting emotional label**.

	**Angry**	**Happy**	**Sad**	**Fearful**	**Neutral**
Movie clip 1: sad	1	2	91	1	5
Movie clip 2: happy	0	86	2	1	11
Movie clip 3: fearful	6	0	2	88	4
Movie clip 4: neutral	2	0	9	0	89
Movie clip 5: neutral	3	1	0	0	96
Movie clip 6: sad	0	0	99	1	0
Movie clip 7: sad	0	0	92	3	5
Movie clip 8: fearful	28	0	1	68	3
Movie clip 9: angry	91	1	0	1	7
Movie clip 10: angry	55	26	4	10	5
Movie clip 11: angry	71	19	1	7	2
Movie clip 12: neutral	0	0	4	1	95
Movie clip 13: neutral	1	10	2	0	87
Movie clip 14: sad	0	2	68	26	4
Movie clip 15: angry	69	1	1	5	24
Movie clip 16: fearful	3	2	1	85	9
Movie clip 17: angry	37	16	5	35	7
Movie clip 18: happy	14	86	0	0	0
Movie clip 19: happy	7	72	0	0	21
Movie clip 20: happy	1	98	0	0	1
Movie clip 21: sad	2	3	83	6	6
Movie clip 22: happy	1	98	1	0	0

**Table 3:** **Scores on the body language reading test (point-light walkers) in participants with schizophrenia (SZ) and healthy participants (HC) across sex**.

	**SZ**			**HC**			**Statistics**
	**Total *n* = 84**	**Males *n* = 53**	**Females *n* = 31**	**Total *n* = 84**	**Males *n* = 51**	**Females *n* = 33**	
EmoBio total	0.79 (0.14)	0.79 (0.14)	0.80 (0.16)	0.87 (0.08)	0.86 (0.08)	0.88 (0.09)	ANOVA: Group: *F* = 16.68^**^
EmoBio angry	0.73 (0.25)	0.73 (0.25)	0.72 (0.25)	0.81 (0.17)	0.81 (0.15)	0.81 (0.21)	ANOVA: Group: *F* = 15.19^**^ Sex: *F* = 0.97, ns Emotion: *F* = 13.32^**^ All interaction effects: ns
EmoBio happy	0.83 (0.18)	0.82 (0.16)	0.86 (0.19)	0.91 (0.11)	0.92 (0.09)	0.89 (0.13)	
EmoBio sad	0.81 (0.21)	0.79 (0.22)	0.85 (0.19)	0.87 (0.17)	0.85 (0.19)	0.90 (0.12)	
EmoBio fearful	0.71 (0.32)	0.71 (0.30)	0.71 (0.36)	0.82 (0.18)	0.82 (0.16)	0.82 (0.21)	
EmoBio neutral	0.86 (0.17)	0.86 (0.18)	0.86 (0.17)	0.92 (0.13)	0.90 (0.12)	0.96 (0.12)	

*EmoBio, Emotional Biological Motion; ns, non-significant*.

** *p < 0.001*.

### Statistical analyses

Analyses were done using The Statistical Package for the Social Sciences (IBM SPSS Statistics for Windows, Version 22.0, IBM Corp, Armonk, NY). Normality of distributed scores was investigated through histograms and skewness indices. All EmoBio measures had negative skewness values with scores clustering at the high end. The Kolmogorov-Smirnov statistic was significant (*p* < 0.001) for all measures. Only one outlier was identified, and only for one of the measures (EmoBio happiness). Although a skewed distribution is expected for this kind of stimuli and only one extreme value was present in our dataset, a cautious approach was chosen and the data transformed. As the data was negatively skewed they were reflected before undergoing logarithmic transformations (log10). The outlier participant was not excluded from analyses. An initial univariate analysis of variance ANOVA looked at overall case-control differences for the total EmoBio score. Thereafter, a 2 × 2 × 5 repeated measures ANOVA (also known as mixed between-within subjects ANOVA) of the effect of diagnostic group (healthy control participants/schizophrenia participants) and sex (male/female) on body language reading was conducted. The five EmoBio scores (angry, happy, sad, fearful, neutral) were entered as dependent variables (within-subjects factor). Diagnostic group (schizophrenia participants or healthy controls) and sex (male or female) were the between-subject factors. Associations between body language reading and symptoms (PANSS positive and negative symptom scales, respectively, and GAF-s) and functioning (GAF-f) were investigated using bivariate correlations (Pearson's *r*) for the 80 individuals with schizophrenia where such data existed.

## Results

There was a significant difference between healthy controls and schizophrenia participants for the total EmoBio score [*F*_(1, 166)_ = 16.68, *p* < 0.001, η^2^ = 0.09]. The Levene test statistic (15.17, *p* < 0.001) indicated unequal variances across groups, but the robust Welch test for equality of means (16.68, *p* < 0.001) still showed highly significant group differences. The effect size (Cohen's *d*) using the pooled standard deviation was 0.73. The diagnostic group difference remained significant after controlling for intellectual abilities as a covariate [*F*_(1, 167)_ = 6.92, *p* = 0.009, η^2^ = 0.04]. Therefore, and because compromised intellectual abilities are an inherent feature of schizophrenia, this was not controlled for in subsequent analyses as this can be conceived of as controlling the illness away. There were no significant group differences for age or sex distribution.

**Figure 1:**
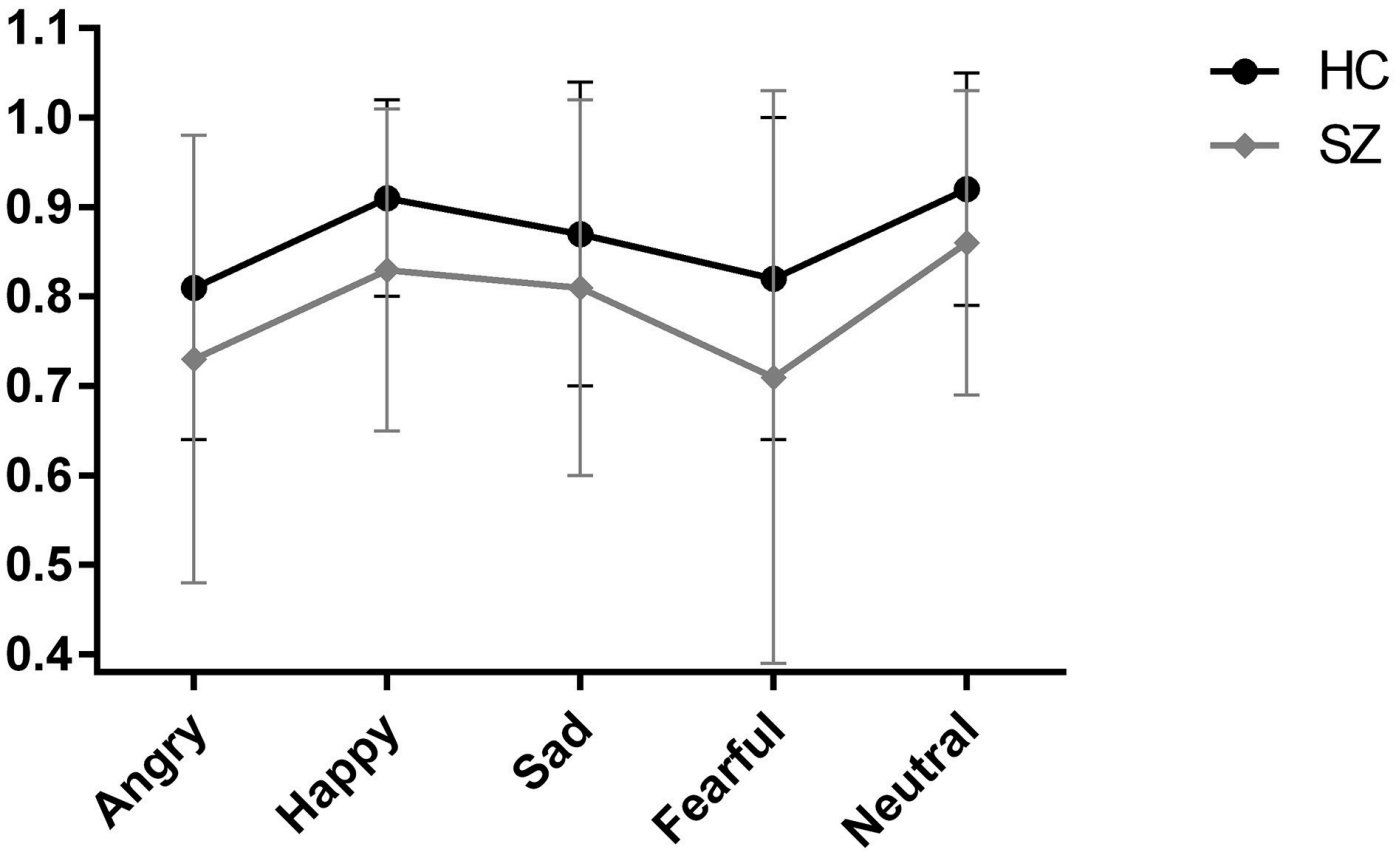
**Scores on the body language reading test of sad, fearful, happy, angry, and neutral point-light walkers in participants with schizophrenia (SZ) and healthy participants (HC)**. Error bars correspond to standard deviations for the two groups.

In the repeated measures ANOVA across the five emotions, the main between-subject effect of diagnostic group was significant [*F*_(1, 164)_ = 15.19, *p* < 0.001, η^2^ = 0.09], with participants with schizophrenia performing significantly worse than healthy control participants, corroborating the results of the first ANOVA for the total score. Further, the main within-subject effect of emotion was significant [*F*_(4, 161)_ = 13.32, Wilk's Lambda = 0.75, *p* < 0.001, η^2^ = 0.25], indicating that performance differed across emotion. This can be seen in Figure 1 where the performance of the two groups (healthy control participants and schizophrenia participants) is depicted. The group × emotion interaction effect was non-significant [*F*_(4, 161)_ = 0.25, Wilk's Lambda = 0.98, *p* = 0.521, η^2^ = 0.02], reflected in the similar profile shape of body language reading performance for healthy control participants and schizophrenia participants in Figure 1. This indicates that schizophrenia participants are not disproportionally impaired on any one emotion compared to healthy control participants. Neither the main between-subject effect of sex [*F*_(1, 164)_ = 0.97, *p* = 0.327, η^2^ = 0.01], nor any of the other interaction effects were significant.

Bivariate associations between body language reading and measures of symptoms or functioning in the 80 participants with schizophrenia where the latter data was available were non-significant. The strongest, albeit small, correlation was found for positive symptoms assessed with the PANSS (*r* = −0.12, *p* = 0.308).

## Discussion

In this study we found that persons with schizophrenia were impaired in overall body language reading compared to healthy controls. The magnitude of this impairment was medium-large (*d* = 0.73) and similar to what was found in an American study (*d* = 0.69) (Kern et al., 2013). This is also in line with what we know about emotion perception (*d* = 0.91) (Kohler et al., 2010) and other domains of social cognition (Savla et al., 2013) in schizophrenia with scores markedly below healthy comparison groups. The body language reading impairment did not depend on sex or on which emotion was expressed. The deficit in body language reading in schizophrenia therefore seems to be of a global or general nature.

Our second research aim concerned whether some emotions were easier to recognize than others. For both groups, performance differed across emotions, suggesting that some emotions in fact were easier to read from body language than others. Neutral and happy motion in PLD walkers was most easily recognizable, whereas decoding fear and anger from such stimuli appears to be more difficult (see Figure 1). The pattern seen in healthy participants and participants with schizophrenia were identical. Results stand in contrast to previous studies of PLDs in healthy control participants (Chouchourelou et al., 2006; Ikeda and Watanabe, 2009) where sensitivity to anger was greater than sensitivity to other emotions. However, the results align with results for facial emotion perception. Research in healthy normal persons has found facial expressions of happiness to be the easiest and fear the hardest emotion to recognize (Montagne et al., 2007). In other words, when it comes to specific emotions, our work corroborates findings from the facial emotion perception literature, but not the limited body language literature. There are a few possible explanations. The few previous studies that looked at differences in the ability to recognize specific emotions had very small sample sizes (Chouchourelou et al., 2006; Ikeda and Watanabe, 2009), suggesting that earlier findings of enhanced recognition of anger from PLDs are only preliminary. Alternatively, the patterns, or profile, across emotions could be due to the psychometric properties of the different instruments used in this field. If that is the case, the recognition patterns across emotions are not due to “true” differences between emotions, but instead attributable to differences in task demand. For instance, we cannot rule out that differences across emotions is due to less clarity for some of the PLD movie clips, i.e., that the reference sample did not agree completely on which emotion was displayed.

For our participants with schizophrenia, deficits in body language reading were present for all emotions, and they were not larger for fear, or any other emotion. It therefore diverges from the findings of previous study using the same EmoBio stimuli (Henry et al., 2010) where reduced performance compared to healthy controls only appeared for fear. It also stands in contrast to findings from the facial emotion perception literature. For early onset and first episode psychosis (reviewed by Barkl et al., 2014) there may be larger impairments for some emotions, among them fear (and surprise, disgust) than others (happiness, sadness) and no impairments for neutral and angry stimuli. For established schizophrenia, recognition of facial expressions of fear might be selectively impaired (Edwards et al., 2002). However, our results are in line with most studies that investigated body language reading (Tomlinson et al., 2006; Van den Stock et al., 2011), including one study that used the same stimuli (Couture et al., 2010). Another recent study (Strauss et al., 2015), using dynamic whole-body stimuli, found specific deficits for happiness and sadness. Follow-up one-way ANOVAs yielded statistically significant group differences for two of four emotions, apparently indicating that schizophrenia participants have a specific impairment in body language reading for these two emotions. It should be noted, however, that the overall group × emotion interaction effect in that study was non-significant (Strauss et al., 2015), which is exactly the same as we found in the present study. A cautious interpretation of the results, and of a non-significant interaction effect, is that persons with schizophrenia were not disproportionately impaired on any one emotion compared to healthy control participants. With that interpretation, there is in fact only one study that has found a specific impairment in body language reading for persons with schizophrenia (Henry et al., 2010). We doubt that persons with schizophrenia only have body language reading impairment for one of the emotions, such as fear. There are two reasons for this. First, because our study is large, including a healthy control group recruited through national statistical records, we have the power to pick up relevant effects. When this is not the case, it is less likely that group differences for the perception of specific emotions actually are present. Second, as our overall effect size for group differences in body language reading is very similar to the effect size in a large American study using the same stimuli (Kern et al., 2013), there is reason to believe that significant group differences are present for all emotions, even in that study. Further, a meta-analysis of social cognition has found large deficits for all social cognitive domains in schizophrenia (Savla et al., 2013); making it unlikely that body language reading should differ substantially from this picture.

The third research aim of the current study investigated whether there were sex differences in body language reading. We found no overall significant sex difference in reading emotions from body language, nor did we find any significant “different sex differences” in the schizophrenia sample compared to healthy control participants. Although in contrast to the only study that has investigated the effect of body language reading in schizophrenia (Strauss et al., 2015), there are in fact good arguments for why this might be so. As part of the criticism that “neurosexism,” i.e., behavioral differences between males and females are explained solely by referring to their brains being different, is taking place within neuroscience (Fine, 2014), it is argued that overlap between males and females in a number of characteristics are in fact substantial, and that male-female differences vary over time, place and context. Consequently, sex effects will sometimes be significant, other times not. Also, as has been pointed out by scholars calling for a paradigm shift within research on sex, behavior and psychopathology (Joel and Yankelevitch-Yahav, 2014); biological sex always interacts with factors such as genetic variation and environmental factors to affect brain and behavior. In our case, the negative effect of schizophrenia on body language reading probably overrides any effect of sex on the same process.

Within the schizophrenia group, body language reading ability did not have symptomatic and functional correlates. The lack of associations with symptoms is expected, as social cognition is generally regarded as a separate symptom dimension, not reducible to the positive and negative symptoms of schizophrenia. The strongest association was found with positive symptoms. This is in line with findings a previous study using the same measure (Olbert et al., 2013), as was the size of the correlation coefficient (*r* = -0.10 vs. -0.12 in our study). Based on the literature on social cognition as a predictor of functioning in schizophrenia (Fett et al., 2011), the lack of a significant association with functioning is somewhat surprising. However, the use of a crude measure of functioning, the GAF-f score, could be responsible for this. An alternative explanation is that our measure of social cognition is taken from social neuroscience. Therefore, its placement in a causal pathway from neurobiology to functioning (see Green et al., 2012 for an example of such a pathway) may be earlier than is the case for other measures of social cognition, and therefore less strongly associated with functioning. The limited association with functioning is in line with Olbert et al.'s (2013) results. They found stronger associations with functional capacity than with real-world functioning. The former is generally considered to be closer to neurobiology. Therefore, we encourage the use of more sophisticated measures of functioning and functional capacity in future studies, as it would be somewhat surprising if impaired body language reading ability did not have functional consequences, particularly in the social realm.

One limitation of our study is that we used stimuli where the psychometric properties of the individual items (emotions) are unknown. Also, the study would also have benefited from the inclusion of more detailed measures of functional outcome. Strengths of the study include the use of a large, representative sample of both healthy control and schizophrenia participants, and the adherence to a careful statistical approach in order to avoid Type I errors.

In summary, we found a global impairment in body language reading in schizophrenia that was present for all emotions and in both males and females.

## Funding

This work was supported by the South-Eastern Norway Regional Health Authority (grant No. 2010007 to AV and grant No. 2013123), the Fulbright Foundation for Educational Exchange (to AV), the Research Council of Norway (grant No. 223273) and the K.G. Jebsen Foundation.

### Conflict of interest statement

The authors declare that the research was conducted in the absence of any commercial or financial relationships that could be construed as a potential conflict of interest.
